# Prostate-specific antigen testing patterns and prostate cancer stage at diagnosis in older Ohio cancer patients

**DOI:** 10.1007/s10552-024-01908-x

**Published:** 2024-09-03

**Authors:** Sajan N. Patel, Long Vu, Holly E. Hartman, Weichuan Dong, Siran M. Koroukian, Johnie Rose

**Affiliations:** 1 Department of Population and Quantitative Health Sciences, Case Western Reserve University School of Medicine, Cleveland, OH USA; 2 Case Comprehensive Cancer Center, Case Western Reserve University School of Medicine, Cleveland, OH USA; 3 Center for Community Health Integration, Case Western Reserve University School of Medicine, Cleveland, OH USA

**Keywords:** Prostate-specific antigen testing, Screening, Diagnostic testing, Prostate cancer, Frailty

## Abstract

**Background:**

Prostate cancer (PCa) screening recommendations do not support prostate-specific antigen (PSA) screening for older men. Such screening often occurs, however. It is, therefore, important to understand how frequently and among which subgroups screening occurs, and the extent of distant stage PCa diagnoses among screened older men.

**Methods:**

Using the 2014–2016 linked Ohio Cancer Incidence Surveillance System (OCISS) and Medicare administrative database, we identified men 68 and older diagnosed with PCa and categorized their PSA testing in the three years preceding diagnosis as screening or diagnostic. We conducted multivariable logistic regression analysis to identify correlates of screening PSA and to determine whether screening PSA is independently associated with distant stage disease.

**Results:**

Our study population included 3034 patients (median age: 73 years). 62.1% of PCa patients underwent at least one screening-based PSA in the three years preceding diagnosis. Older age (75–84 years: aOR [95% CI]: 0.84 [0.71, 0.99], ≥ 85: aOR: 0.27 [0.19, 0.38]), and frailty (aOR: 0.51 [0.37, 0.71]) were associated with lower screening. Screening was associated with decreased odds of distant stage disease (aOR: 0.55 [0.42, 0.71]). However, older age (75–84 years: aOR: 2.43 [1.82, 3.25], ≥ 85: aOR: 10.57 [7.05, 15.85]), frailty (aOR: 5.00 [2.78, 9.31]), and being separated or divorced (aOR: 1.64 [1.01, 2.60]) were associated with increased distant stage PCa.

**Conclusion:**

PSA screening in older men is common, though providers appear to curtail PSA screening as age and frailty increase. Screened older men are diagnosed at earlier stages, but the harms of screening cannot be assessed.

## Introduction

Prostate cancer (PCa) is the second most common cancer among the United States men [1]. Although the incidence of PCa has remained stable over the past decade, the percentage of PCa cases diagnosed at a distant stage has increased from 3.9 to 8.2% over the same time span [2]. Elevated Prostate-Specific Antigen (PSA) level is a relatively sensitive, although not highly specific, marker of PCa [3]. When prostate-specific antigen (PSA) levels are abnormally high, further testing, such as a biopsy, may be necessary to ascertain PCa diagnosis [4].

The United States Preventive Services Task Force (USPSTF) issues and periodically revises recommendations regarding the use of PSA screening for PCa detection. In 2008, the USPSTF recommended against screening for PCa for men aged 75 years and older and stated that there was insufficient evidence to assess the benefits and harms of screening for those younger than 75 years [5]. By 2012, the USPSTF changed its recommendation to advise against PSA screening for men of any age [6]. In the most recent recommendations issued in 2018, the USPSTF recommended that screening for men aged 55 to 69 years should be an individual decision made by men in partnership with their medical provider [7]. Similarly, the American Urological Association (AUA) recommends shared decision-making by men between ages 55 and 69 and their providers [8]. Overall, both USPSTF and AUA recommendations largely discourage PSA screening for men 70 and older [7, 8].

PCa screening practices in the real world frequently differ from clinical recommendations [9, 10]. Screening utilization among older men continues to remain high. In 2022, 32.2% of men 70 years and older on Medicare preferred provider organization plans were still utilizing screening-based PSA against medical advice [11]. With this knowledge, it is important to understand who among the older male population is getting screened and how screening impacts future PCa diagnosis. Juxtaposed in the decision-making process is the evidence that regular PSA-based screening in clinical practice has increased the likelihood of diagnosing PCa at an early stage [12], but that PSA screening likely contributes to overdiagnosis of cases of PCa which will not themselves prove fatal [13].

In this study, we examined patterns of PSA testing for screening versus diagnostic purposes according to age, frailty, and other factors among Medicare enrolled men. We also explored the association of screening PSA use with stage at diagnosis. Identifying widespread screening among older men unlikely to benefit may help reduce the harms of overscreening. At the same time, there is utility in understanding whether screening, in a real-world setting, is associated with decreased metastatic disease among older men when controlling for confounders associated with PCa staging.

## Materials and methods

### Data sources and study population

We used data from the 2014–2016 Ohio Cancer Incidence Surveillance System (OCISS) and Medicare linked database. The OCISS is supported and certified by both the National Program of Cancer Registries and North American Association of Central Cancer Registries [14]. Maintained by the Ohio Department of Health, the OCISS collects information on demographics, patient’s residence address, insurance status, cancer site, stage, tumor size, and histology [14]. OCISS data were linked with Medicare claims data by the Centers for Medicare & Medicaid Services (DUA 2012–23469). This study was approved by the Case Western Reserve University Institutional Review Board (Protocol #20,120,107). This integrated OCISS-Medicare database included data for the subpopulation of cancer patients who were also Medicare beneficiaries, and received their care through the traditional fee-for-service system to ensure complete claims history.

**Fig. 1:**
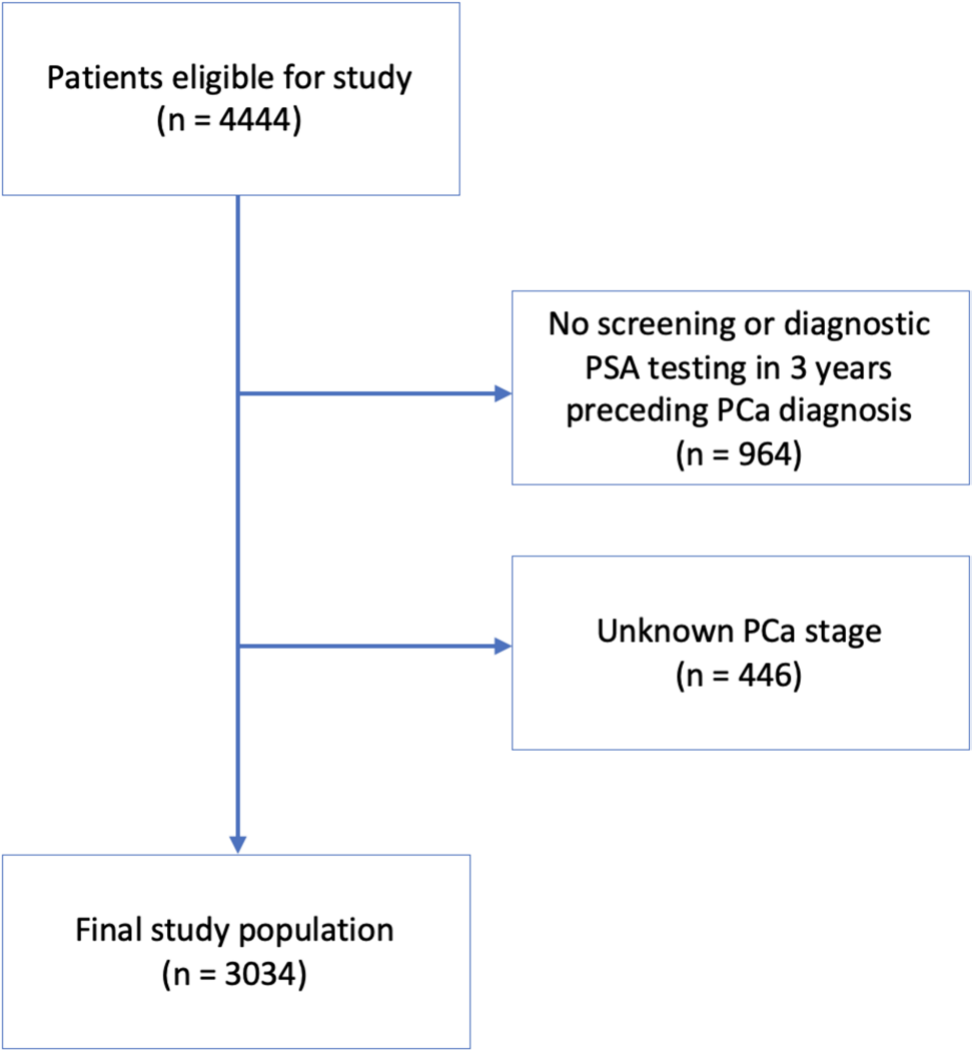
Flowchart showing patient selection for study population

The study population included older men residing in the State of Ohio who received a PCa diagnosis from 2014 to 2016. PCa patients were identified using tumor site data from OCISS. In order to allow for the analysis of PSA testing patterns in the three years preceding cancer diagnosis, we limited our study population to those who received a PCa diagnosis at the age of 68 years or older. We excluded individuals whose PCa diagnosis was documented in the OCISS, but for whom there was no screening or diagnostic PSA testing information from Medicare claims data in the three years prior to diagnosis (*n* = 964). A washout period of 30 days after the date of diagnosis as reported from the OCISS was accounted for to capture additional PSA tests that may have been received around the same time period of the diagnosis. We also excluded patients who were unstaged at diagnosis (*n* = 446). Our final study population included 3034 men (Fig. 1).

### Variables of interest

Demographic variables included age at the time of PCa diagnosis, collapsed into three categories—68–74 years old, 75–84 years old, and 85 years or older, and race, collapsed into two categories—White and non-White, given the small number of men falling into other racial categories. Additionally, we included a five-category variable defining marital status at the time of diagnosis—married, separated/divorced, widowed, single (never married), and unknown. Other variables included geographic area (metropolitan, Appalachian, or non-Appalachian and non-metropolitan [rural]) and neighborhood poverty level derived from US census tract data (< 5%, 5–9.99%, 10–19.99%, or ≥ 20%) to gain a better perspective of each patient’s environment.

Patient frailty, a key independent variable associated with higher risk of death, hospitalization, falls, and healthcare utilization, was operationalized using a validated deficit-accumulation frailty index (claims-based frailty index) [15]. In addition to capturing comorbidities, frailty more completely captures a patient’s functional status and vulnerability to adverse outcomes [16]. The claims-based frailty index (CFI) was calculated based on one year of Medicare claims data prior to PCa diagnosis. These scores were broken into three frailty categories. Patients with a CFI less than 0.1 were classified as non-frail, those with a CFI between 0.10 and 0.19 were classified as pre-frail, and patients greater than 0.20 were classified as mild or frailer. The first outcome of interest was the indication for PSA testing received—diagnostic or screening, operationalized by adapting an existing algorithm to group colonoscopy, sigmoidoscopy, and fecal occult blood test as screening, diagnostic, or surveillance testing [17]. Diagnosis codes V76.44 (ICD-9-CM) or Z12.5 (ICD-10-CM) were used for encounters for malignant neoplasm of the prostate through yearly screening. If one of these ICD codes was present, the patient was classified as having received a screening PSA. Similarly, HCPCS code G0103 (prostate cancer screening; PSA test) was considered indicative of screening PSA. A claim for which one of these codes was absent and the CPT codes 84,152 (PSA; completed) and 84,153 (PSA; total), 84,154 (PSA; free) were present was classified as diagnostic PSA. After documentation of three screening tests during the three-year lookback period, any additional testing was classified as diagnostic because Medicare beneficiaries are only covered for annual screening PSA. The secondary outcome of interest was SEER Summary Stage of PCa at diagnosis—localized, regional, and distant [18].

### Analytic approach

In addition to descriptive analysis, we conducted logistic regression analysis to identify correlates of receipt of screening PSA and subsequently, to determine whether receipt of screening PSA was associated with distant stage at diagnosis after accounting for potential confounders including age, race, area poverty level, marital status, geographic area, and frailty. Analysis was conducted using SAS version 9.4 and R 4.1.1 and 4.2.2.

## Results

From the study population (*n* = 3034), 1884 (62.1%) patients underwent one or more screening PSA tests in the three years preceding PCa diagnosis (Table 1). More men with receipt of a screening PSA were between 68 and 74 years old compared to those who received diagnostic PSA testing (66.8% vs. 57.7%). A larger percentage of patients with only screening PSA were married compared to those who underwent diagnostic PSA testing (73.1% vs. 69.2%). Race, geographic area classification, and area poverty level were comparable between the screening and diagnostic groups. A higher proportion of men who underwent only screening PSA were non-frail compared to those who received diagnostic testing (23.7% vs. 16.0%), while a smaller percentage of screening-based PSA patients were mildly frail or frailer (5.8% vs. 9.1%). Men who underwent diagnostic PSA testing had higher rates of distant stage PCa diagnosis (14.3% vs. 6.4%).

**Table 1:** Study population characteristics comparing screening and diagnostic PSA test groups

	Total count	Screening (*n* = 1884)	Diagnostic (*n* = 1150)
Variables		N (col %)	N (col %)
Number of patients			
	3034	1884 (62.1)	1150 (37.9)
Age at diagnosis (in years)			
68–74	1923	1259 (66.8)	664 (57.7)
75–84	945	571 (30.3)	374 (32.5)
≥ 85	166	54 (2.9)	112 (9.7)
Race			
White	2686	1668 (88.5)	1018 (88.5)
Non-white	348	216 (11.5)	132 (11.5)
Marital status			
Married	2174	1378 (73.1)	796 (69.2)
Separated/divorced	194	119 (6.3)	75 (6.5)
Widowed	265	153 (8.1)	112 (9.7)
Single	237	144 (7.6)	93 (8.1)
Unknown	164	90 (4.8)	74 (6.4)
Geographic area			
Metropolitan	2005	1239 (65.8)	766 (66.6)
Appalachian	524	335 (17.8)	189 (16.4)
Rural	505	310 (16.5)	195 (17.0)
Area poverty level			
< 5%	899	538 (28.6)	361 (31.4)
5.00–9.99%	812	518 (27.5)	294 (25.6)
10.00–19.99%	836	539 (28.6)	297 (25.8)
≥ 20%	487	289 (15.3)	198 (17.2)
Claims-based frailty index			
Non-frail (score < 0.1)	630	446 (23.7)	184 (16.0)
Pre-frail (0.1–0.19)	2189	1328 (70.5)	861 (74.9)
Mildly-severely frail (score ≥ 0.20)	215	110 (5.8)	105 (9.1)
Stage of prostate cancer diagnosis			
Localized	2358	1512 (80.3)	846 (73.6)
Regional	390	251 (13.3)	139 (12.1)
Distant	286	121 (6.4)	165 (14.3)

**Fig. 2:**
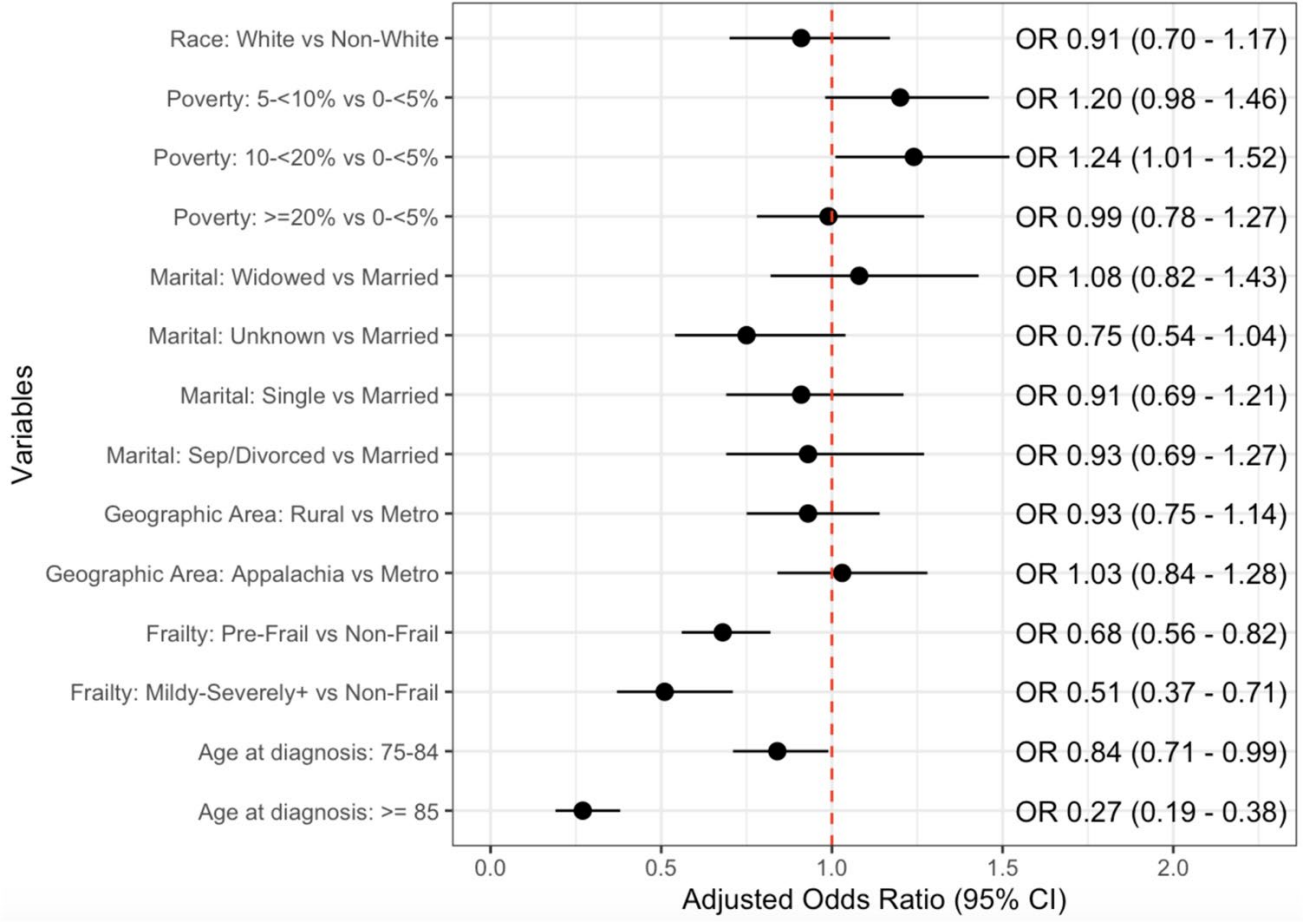
Adjusted odds ratios from logistic regression model predicting receipt of screening PSA test

Results from the logistic regression analysis showed no association between screening and race, poverty level, marital status, and geographic area (Fig. 2). However, receipt of screening PSA was significantly associated with frailty and age at diagnosis. Compared to non-frail men, those who were classified as pre-frail had significantly lower odds of undergoing a screening PSA test (adjusted odds ratio (aOR): 0.68 [95% confidence interval: 0.56, 0.82]). When comparing mild and frailer patients to non-frail, the odds of receiving a screening PSA test decreased further (aOR: 0.51 [0.37, 0.71]). With respect to men between 68 and 74 years old at diagnosis, men between 75 and 84 years old (aOR: 0.84 [0.71, 0.99]) and 85 years or older (aOR: 0.27 [0.19, 0.38]) had significantly reduced odds of undergoing a screening PSA test.

Differences in stage at diagnosis when comparing against several demographic factors, frailty, and receipt of screening PSA were assessed in Table 2. A larger proportion of men diagnosed with PCa at a distant stage were 85 years old or older compared to those diagnosed with localized or regional disease (25.2% vs. 3.4%). Race, geographic area, and area poverty level did not significantly vary across stage categories. With respect to marital status, patients diagnosed at a distant stage compared to a localized or regional stage were more likely to be widowed (17.8% vs. 7.8%), while earlier-stage PCa patients were more likely to be married (72.9% vs. 60.1%). Patients diagnosed with distant stage PCa were less likely to be non-frail (6.3% vs. 22.3%) and more likely to exhibit mild to severe frailty (16.1% vs. 6.1%). Participation in screening-based PSA testing was more likely to occur in patients diagnosed at an early stage compared to advanced (63.3% vs. 42.3%), while participation in diagnostic testing was more likely to occur in patients diagnosed at a distant stage (57.7% vs. 36.7%).

**Table 2:** Comparing patient information between distant and localized/regional stage diagnoses

	Total Count	Distant (*n* = 286)	Localized/Regional (*n* = 2748)
Variables		N (col %)	N (col %)
Number of PATIENTS			
	3034	286 (9.4)	2748 (90.6)
Age at diagnosis (in years)			
68–74	1923	95 (33.2)	1828 (66.5)
75–84	945	119 (41.6)	826 (30.1)
≥ 85	166	72 (25.2)	94 (3.4)
Race			
White	2686	248 (86.7)	2438 (88.7)
Non-white	348	38 (13.3)	310 (11.3)
Marital status			
Married	2174	172 (60.1)	2002 (72.9)
Separated/divorced	> 193*	> 24 (> 8.4)*	< 170 (> 6.2)*
Widowed	265	51 (17.8)	214 (7.8)
Single	237	28 (9.8)	209 (7.6)
Unknown	< 165*	< 11 (< 3.8)*	> 153 (> 5.6)*
Geographic area			
Metropolitan	2005	189 (66.1)	1816 (65.2)
Appalachian	524	51 (17.8)	473 (17.0)
Rural	505	46 (16.1)	459 (16.5)
Area poverty level			
< 5%	899	75 (26.2)	824 (29.6)
5.00–9.99%	812	72 (25.2)	740 (26.6)
10.00–19.99%	836	82 (28.7)	754 (27.1)
≥ 20%	487	57 (19.9)	430 (15.4)
Claims-based frailty index			
Non-frail (score < 0.1)	630	18 (6.3)	612 (22.3)
Pre-frail (0.1–0.19)	2189	222 (77.6)	1967 (71.6)
Mildly-severely frail (score ≥ 0.20)	215	46 (16.1)	169 (6.1)
PSA testing in 3 years prior to cancer diagnosis			
Screening	1884	121 (42.3)	1763 (63.3)
Diagnostic	1150	165 (57.7)	985 (36.7)

*In accordance with our data users’ agreement, cell sizes < 11 were masked to prevent re-identification of subjects. Other cells in the corresponding rows and columns were masked to prevent the derivation of the masked cells

**Fig. 3:**
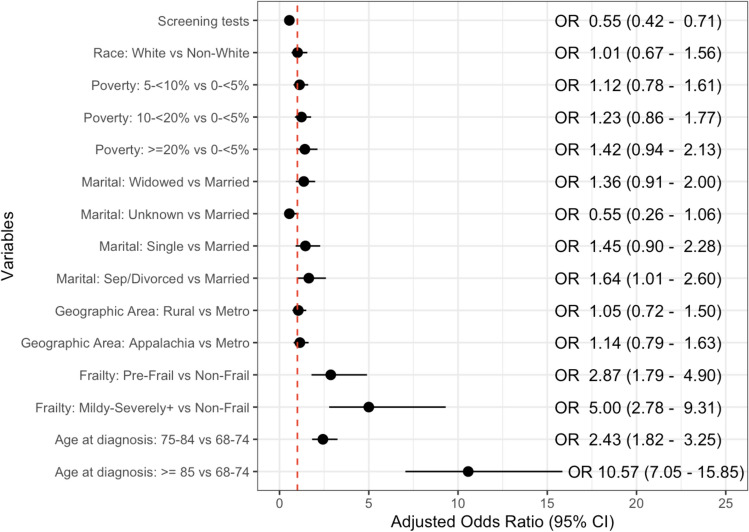
Adjusted odds ratios from logistic regression model predicting distant stage prostate cancer diagnosis

The multivariable logistic regression model predicted reduced odds of distant stage PCa diagnosis for patients receiving screening PSA (aOR: 0.55 [0.42, 0.71]) (Fig. 3). Race, geography, and area poverty level were not independently associated with distant stage diagnosis. Divorced or separated patients, as opposed to married, had increased odds of distant stage disease (aOR: 1.64 [1.01, 2.60]). A pre-frail classification compared to non-frail increased the predicted odds of distant stage diagnosis (aOR: 2.87 [1.79, 4.90]). The odds ratio of distant staging for mild to severely frail patients compared to a reference of non-frail patients increased to 5.00 (95% CI: 2.78, 9.31). Additionally, men between 75 and 84 years old at the time of diagnosis (aOR: 2.43 [1.82, 3.25]) and men 85 years or older (aOR: 10.57 [7.05, 15.85]), compared to those between 68 and 74 years old, were associated with elevated odds of distant stage diagnosis.

## Discussion

The objective of this study was to understand PSA testing patterns for older prostate cancer patients during the three years prior to their PCa diagnosis, and whether receipt of screening PSA testing was associated with distant stage disease at diagnosis. We used the OCISS-Medicare linked database to capture these trends among Ohio prostate cancer patients aged 68 years and older who had undergone at least one PSA test. 62.1% of PCa patients underwent at least one screening-based PSA in the three years preceding diagnosis. Patients with mild or greater frailty (aOR: 0.51 [0.37, 0.71]) had significantly lower odds of undergoing PSA screening. Additionally, men between 75 and 84 years old at diagnosis (aOR: 0.84 [0.71, 0.99]) and 85 years or older (aOR: 0.27 [0.19, 0.38]) were associated with lower odds of receiving a screening PSA compared to men between 68 and 74 years old. Receipt of screening PSA tests was independently associated with a decrease in the odds of distant stage diagnosis (aOR: 0.55 [0.42, 0.71]). However, men between 75 and 84 years old at the time of diagnosis (compared to men 68–74 years old) (aOR: 2.43 [1.82, 3.25]), 85 years or older (compared to men 68–74 years old) (aOR: 10.57 [7.05, 15.85]), having moderate to severe frailty (compared to non-frailty) (aOR: 5.00 [2.78, 9.31]), and being separated or divorced (compared to being married) (aOR: 1.64 [1.01, 2.60]) were independently associated with increased odds of distant stage PCa diagnosis.

Screening practices seen in the real world only partially align with current clinical guidelines. The odds for participating in screening significantly lowered for individuals between 75 and 84 and even more so in the population of men 85 years or older. This aligns with the most recent guidelines from the USPSTF and AUA that discourage any participation in screening over the age of 69 [7, 8]. This is beneficial to patients due to the diminished utility of screening at older age due to negative effects of overdiagnosis and overtreatment of PCa. That being said, it is noteworthy that 62.1% of PCa patients in this study received at least one screening-based PSA, given that the study population consisted of individuals 68 or older to allow for ascertainment of receipt of PSA testing in the three years preceding their diagnosis. This finding provides further evidence [11] of a discordance between real-world testing and provider recommendations.

Our modeling approaches indicated that frailer patients were less likely to receive screening PSA compared to their non-frail counterparts, which aligns with results from prior literature. Another study of men older than 50 years that aimed to test factors influencing uptake of PSA testing found that greater frailty was associated with a lower likelihood of undergoing PSA screening in a situation deemed as low value [19]. In that study, frailer individuals would be more likely to present with other comorbidities and would subsequently complete diagnostic testing as opposed to screening.

Additionally, our study showed statistically significantly lower odds of distant stage prostate cancer diagnosis associated with PSA screening. These findings align with those of Etzioni et al. who concluded from their modeling study that PSA screening accounted for much of the observed decrease in distant stage PCa incidence [20]. That being said, we were unable to assess the harms and benefits of early-stage diagnosis for these patients. Although screening may offer benefits to some patients, it is important to recognize that overscreening can cause more harm than good in many instances, especially among older men in the forms of overdiagnosis and overtreatment [21]. As previously mentioned, the usage of PSA as a measure to indicate the presence of PCa is a relatively sensitive, but not specific test, which can lead to increased false positive cases. Additionally, prostate cancer’s variable disease progression results in a significant portion of patients detected with and treated for prostate cancer through PSA screening who are likely to die of a different cause before the presence of any clinical manifestations of PCa [21] and can include invasive prostate biopsies and cause side effects, including erectile dysfunction, urinary incontinence, bowel dysfunction, and death in severe cases [22]. Individuals treated for PCa can often face a diminished quality of life as well as a financial burden due to increased utilization of health services [22].

With respect to marital status, our results showed that individuals separated or divorced faced higher risk of distant stage disease compared to their married counterparts. This finding aligns with a study conducted by Abdollah et al. to determine the relationship between marital status, stage, and survival of prostate cancer as they found men who were separated, divorced, or widowed were more likely to have advanced stage cancer at the time of surgery [23].

The choice of the OCISS-Medicare database allowed for a greater capture of both clinical and demographic data that could not have been achieved with using these sources alone. This allowed for the inclusion of many predictors, ranging from demographic variables such as race and geographic area (at a far more granular level than SEER-Medicare can provide) to clinical information, such as the number of PSA tests a patient received. Additionally, usage of the OCISS-Medicare database allowed for examination for outcomes in a state, Ohio, that is not a part of the SEER-Medicare database. A limitation in this study is that unstaged cancer diagnoses were excluded. Ten percent of PCa patients who met the inclusion criteria for the study from the OCISS-Medicare database were not staged at diagnosis. It is unlikely that these cases occurred at random, as research suggests that vulnerable populations such as racial minorities, those in need of complex care, uninsured individuals, and people with lower educational attainment are more likely to have an unstaged cancer diagnosis [24]. Additionally, there appeared to be variability in coding practices for PSA tests as the initial population captured had nearly 20% of prostate cancer patients for whom no PSA test was recorded, leading us to restrict the population further to only capture individuals documented to have undergone at least one PSA test. Differences in coding practices across health care organizations could have also resulted in misclassification of testing. Only 62% of patients coded as having received diagnostic PSA had one or more PCa-related symptoms coded in relation to the testing. However, we chose a conservative operational definition of screening (leading to likely underestimation of actual screening) since screening is not recommended for the majority of individuals in our study group. Specifically, we only considered a PSA test to be screening if it was explicitly coded as a screening test. With regard to PSA testing, no data in this study were collected on the serum PSA levels, which could have provided a more precise assessment of one’s risk for development of PCa. Furthermore, the generalizability of this study is limited due to the fact that only Ohio prostate cancer patients aged 68 and older are considered, so all conclusions cannot necessarily be applied to the United States population as a whole.

## Conclusion

Findings from this study demonstrate the PSA testing patterns among Ohio Medicare beneficiaries with prostate cancer. Screening was widespread in this older population as more men received PSA tests for screening than for diagnostic purposes, despite screening being discouraged for older men by clinical guidelines. However, it appears that older, more frail patients were screened less frequently, which aligns recommendations of reduced screening in low-value settings. This study provides population-based evidence that screening may be associated with lower likelihood of metastatic disease, even among older men. Although whether this translates to improved survival or quality of life cannot be assessed, these findings can inform the conversation regarding the utility of PSA-based screening for older men.

## Data Availability

The Medicare claims data are used under a data use agreement (DUA) with the Centers for Medicare and Medicaid Services. The Ohio Cancer Incidence Surveillance System (OCISS) data are used under a DUA with the Ohio Department of Health. The DUA’s restrict the sharing of data with other investigators. Investigators wishing to replicate the study would need to enter into agreements with CMS and ODH to obtain and use the data. The linkage of the OCISS and Medicare data sets was performed by the Centers for Medicare and Medicaid Services (CMS).

## Appendix

See Table 3, 4, 5, 6

**Table 3:** Results from unadjusted logistic regression model predicting receipt of screening PSA

Term	Odds ratio	95% Confidence interval
Age at diagnosis: 75–84 vs 68–74	0.81	(0.69, 0.95)
Age at diagnosis: ≥ 85 vs 68–74	0.25	(0.18, 0.35)
Race: white vs non-white	1.00	(0.79, 1.26)
Marital status: separated/divorced vs married	0.92	(0.68, 1.24)
Marital status: widowed vs married	0.79	(0.61, 1.02)
Marital status: single vs married	0.89	(0.68, 1.18)
Marital status: unknown vs married	0.70	(0.51, 0.97)
Geographic Area: Appalachian vs Metro	1.10	(0.90, 1.34)
Geographic area: rural vs metro	0.98	(0.80, 1.20)
Poverty: 5- < 10% vs < 5%	1.18	(0.97, 1.44)
Poverty: 10- < 20% vs < 5%	1.22	(1.00, 1.48)
Poverty: ≥ 20% vs < 5%	0.98	(0.78, 1.23)
Frailty: pre-frail vs non-frail	0.64	(0.52, 0.77)
Frailty: mild or frailer vs non-frail	0.43	(0.31, 0.59)

**Table 4:** Results from logistic regression models predicting receipt of screening PSA only adjusted for single covariates and age at diagnosis

Term	Odds ratio	95% Confidence interval
Age at diagnosis: 75–84 vs 68–74	0.81	(0.69, 0.95)
Age at diagnosis: ≥ 85 vs 68–74	0.25	(0.18, 0.35)
Race: white vs non-white	0.99	(0.78, 1.25)
Age at diagnosis: 75–84 vs 68–74	0.80	(0.68, 0.94)
Age at diagnosis: ≥ 85 vs 68–74	0.25	(0.18, 0.35)
Marital status: separated/divorced vs married	0.9	(0.67, 1.23)
Marital status: widowed vs married	1.03	(0.79, 1.36)
Marital status: single vs married	0.90	(0.68, 1.19)
Marital status: unknown vs married	0.72	(0.52, 1.00)
Age at diagnosis: 75–84 vs 68–74	0.81	(0.69, 0.95)
Age at diagnosis: ≥ 85 vs 68–74	0.26	(0.18, 0.36)
Geographic area: appalachian vs metro	1.05	(0.86, 1.28)
Geographic area: rural vs metro	0.97	(0.79, 1.18)
Age at diagnosis: 75–84 vs 68–74	0.81	(0.69, 0.95)
Age at diagnosis: ≥ 85 vs 68–74	0.25	(0.18, 0.35)
Poverty: 5- < 10% vs < 5%	1.19	(0.98, 1.46)
Poverty: 10- < 20% vs < 5%	1.21	(0.99, 1.47)
Poverty: ≥ 20% vs < 5%	0.97	(0.77, 1.22)
Age at diagnosis: 75–84 vs 68–74	0.85	(0.72, 1.00)
Age at diagnosis: ≥ 85 vs 68–74	0.28	(0.20, 0.39)
Frailty: pre-frail vs non-frail	0.68	(0.56, 0.82)
Frailty: mild or frailer vs non-frail	0.51	(0.37, 0.70)

**Table 5:** Results from unadjusted logistic regression model predicting distant stage prostate cancer at diagnosis

Term	Odds ratio	95% Confidence interval
Screening tests	0.41	(0.32, 0.52)
Age at diagnosis: 75–84 vs 68–74	2.77	(2.09, 3.68)
Age at diagnosis: ≥ 85 vs 68–74	14.74	(10.18, 21.36)
Race: white vs non-white	0.83	(0.58, 1.21)
Marital status: separated/divorced vs married	1.72	(1.08, 2.65)
Marital status: widowed vs married	2.77	(1.95, 3.88)
Marital status: single vs married	1.56	(1.00, 2.35)
Marital status: unknown vs married	0.76	(0.37, 1.39)
Geographic area: appalachian vs metro	1.04	(0.74, 1.42)
Geographic area: rural vs metro	0.96	(0.68, 1.34)
Poverty: 5- < 10% vs < 5%	1.07	(0.76, 1.50)
Poverty: 10- < 20% vs < 5%	1.19	(0.86, 1.66)
Poverty: ≥ 20% vs < 5%	1.46	(1.01, 2.09)
Frailty: pre-frail vs non-frail	3.84	(2.42, 6.48)
Frailty: mild or frailer vs non-frail	9.25	(5.32, 16.77)

**Table 6:** Results from logistic regression models predicting distant stage prostate cancer at diagnosis only adjusted for single covariates and age at diagnosis

Term	Odds ratio	95% Confidence interval
Age at diagnosis: 75–84 vs 68–74	2.70	(2.04, 3.59)
Age at diagnosis: ≥ 85 vs 68–74	12.34	(8.46, 18.01)
Screening tests	0.52	(0.40, 0.68)
Age at diagnosis: 75–84 vs 68–74	2.77	(2.09, 3.68)
Age at diagnosis: ≥ 85 vs 68–74	14.72	(10.17, 21.33)
Race: white vs non-white	0.85	(0.59, 1.27)
Age at diagnosis: 75–84 vs 68–74	2.78	(2.09, 3.69)
Age at diagnosis: ≥ 85 vs 68–74	13.97	(9.47, 20.64)
Marital status: separated/divorced vs married	1.91	(1.18, 3.00)
Marital status: widowed vs married	1.46	(0.98, 2.13)
Marital status: single vs married	1.60	(1.01, 2.47)
Marital status: unknown vs married	0.64	(0.31, 1.22)
Age at diagnosis: 75–84 vs 68–74	2.77	(2.09, 3.68)
Age at diagnosis: ≥ 85 vs 68–74	15.07	(10.39, 21.89)
Geographic area: appalachian vs metro	1.23	(0.87, 1.72)
Geographic area: rural vs metro	1.01	(0.70, 1.43)
Age at diagnosis: 75–84 vs 68–74	2.76	(2.08, 3.66)
Age at diagnosis: ≥ 85 vs 68–74	15.12	(10.43, 21.96)
Poverty: 5- < 10% vs < 5%	1.06	(0.74, 1.51)
Poverty: 10- < 20% vs < 5%	1.26	(0.90, 1.79)
Poverty: ≥ 20% vs < 5%	1.56	(1.06, 2.29)
Age at diagnosis: 75–84 vs 68–74	2.49	(1.87, 3.31)
Age at diagnosis: ≥ 85 vs 68–74	12.35	(8.47, 18.00)
Frailty: pre-frail vs non-frail	3.08	(1.93, 5.24)
Frailty: mild or frailer vs non-frail	5.79	(3.25, 10.70)
